# Recent key advances in human immunodeficiency virus medicine and implications for China

**DOI:** 10.1186/1742-6405-7-12

**Published:** 2010-05-26

**Authors:** Kai Sun, Shuntai Zhou, Ray Y Chen, Myron S Cohen, Fujie Zhang

**Affiliations:** 1 School of Medicine, University of North Carolina, Chapel Hill, North Carolina, USA; 2 Washington University in St. Louis, St. Louis, MO, USA; 3 Division of Treatment and Care, National Center for AIDS/STD Control and Prevention, Chinese Center for Disease Control and Prevention, 27 Nanwei Road, Beijing 100050, PR China; 4 National Institute of Allergy and Infectious Diseases, National Institutes of Health, based at the U.S. Embassy Beijing, No. 55 An Jia Lou Lu, Beijing 100600, PR China; 5 China Medical University, Shengyang, Liaoning, PR China

## Abstract

In this article we summarize several recent major developments in human immunodeficiency virus treatment, prevention, outcome, and social policy change. Updated international guidelines endorse more aggressive treatment strategies and safer antiretroviral drugs. New antiretroviral options are being tested. Important lessons were learned in the areas of human immunodeficiency virus vaccines and microbicide gels from clinical studies, and additional trials in prevention, especially pre-exposure prophylaxis, are nearing completion. Insight into the role of the virus in the pathogenesis of diseases traditionally thought to be unrelated to acquired immunodeficiency syndrome has become a driving force for earlier and universal therapy. Lastly, we review important achievements of and future challenges facing China as she steps into her eighth year of the National Free Antiretroviral Treatment Program.

## Introduction

Antiretroviral therapy (ART) has evolved from monotherapy with zidovudine (AZT) to the use of combination nucleoside reverse transcriptase inhibitors (NRTIs), to triple therapy with highly active antiretroviral therapy (HAART), to today's numerous combinations drawn from 6 classes and 32 drugs, including fixed dose formulations, approved by the United States (US) Food and Drug Administration (FDA) [1]. Treatment goals have also progressed from achieving viral suppression to regimen simplification, to long term durability, and to the present paradigm of treatment as prevention. In the past 2 decades, HIV mortality has dramatically decreased reflecting the success of ART [2,3]. New drugs with fewer side effects and lower pill burden have made long term viral suppression a reality. As death rates related to acquired immunodeficiency syndrome (AIDS) continue to decline in patients receiving treatment, attention has shifted to what have heretofore been considered non-AIDS-related deaths. In this paper we will review some of the most important recent advances in HIV medicine and comment on their significance for the future of HIV treatment and care in China.

## HIV Treatment

### New Strategies

The US Department of Health and Human Services (DHHS) [4] and the World Health Organization (WHO) [5] both released new guidelines in 2009. The overall treatment strategies include earlier initiation of ART, individualized treatment based on comorbidities, and regimen optimization to minimize toxicity and potential for drug resistance.

The new US DHHS guidelines for ART initiation have expanded to include all patients with pregnancy, HIV-associated nephropathy, and hepatitis B virus (HBV) coinfection requiring treatment for HBV, regardless of CD4 count, and in all patients with CD4 <350 cells/mm^3^. In addition, ART is now recommended for all patients with CD4 between 350 and 500 cells/mm^3^. As for patients with CD4 > 500 cells/mm^3^, the panel of experts is evenly split between favoring ART initiation and considering it optional. These changes stem from mounting evidence that earlier ART initiation, even before any significant CD4 drop and immune deficiency symptoms, translates to better immune recovery, better tolerance for side effects, smaller risk for TB reactivation, and reduced HIV and TB transmission on a public health level [6-10]. It should be noted, however, that the "moderate level of evidence" for these benefits comes primarily from observational studies conducted in the US and Europe [9,10] and to a large extent reflects reduced cardiovascular complications and cancer. HPTN052 is a multinational trial of 1750 subjects with HIV infection who have been randomized to receive ART at CD4 >350 cells/mm^3 ^or when CD4 declines to 250 cells/mm^3 ^[11]. The Strategic Timing of Antiretroviral Treatment(START) trial, another international multi-center randomized study, is underway to compare immediate commencement of ART at CD4 >500 cells/mm^3 ^to deferral of ART until CD4 declines below 350 cells/mm^3 ^in terms of morbidity and mortality [12]. These trials will provide stronger evidence than observational studies for the benefits of early therapy in asymptomatic patients with high CD4 counts.

Preferred regimens for ART-naïve patients have also been revised. They now include the integrase inhibitor raltegravir (Isentress), recently approved by the US Food and Drug Administration (FDA) for ART-naïve patients, in combination with the NRTIs tenofovir (TDF) and emtricitabine (FTC) [13]. Three other preferred options include: TDF/FTC in combination with efavirenz (EFV), and with the boosted protease inhibitors (PIs) darunavir/ritonavir (DRV/r) and atazanavir/ritonavir (ATV/r). Due to its effects on cholesterol and the gastrointestinal (GI) system, the boosted PI lopinavir/ritonavir (LPV/r) is now an alternate choice except for pregnant women. These regimens are not without side-effects, as EFV causes CNS symptoms and ATV raises bilirubin levels in select patients. Owing to concerns regarding its efficacy in settings of high baseline viral load (VL) and damage on the cardiovascular system, abacavir (ABC) is now an alternative regimen in the US guidelines but was kept as a preferred NRTI backbone in the recently updated European guidelines [14]. Due to conflicting data [15-18], there is not yet a consensus on this issue.

In contrast to the US DHHS guidelines, the WHO recommendations target resource-limited countries and are more conservative. In the most recent WHO draft guidelines [19], immunologic criterion for initiating ART in adults and adolescents was raised from a baseline CD4 of 200 to 350 cells/mm^3^, regardless of symptoms. However, these guidelines have not been widely adapted in part because of limited drug supplies. To reduce rates of mother to child transmission and improve child survival, treatment to prevent mother to child transmission starting at 14 instead of 28 weeks and continuing through breastfeeding was added as an option. Although ART can be delivered safely without routine monitoring of hematology and biochemistry in resource-limited settings based on the Development of AntiRetroviral Therapy in Africa (DART) study [20], expanded laboratory monitoring of CD4 and VL was advised to guide better the switch to second line therapies.

The WHO now urges replacing the NRTI stavudine (d4T) with AZT or TDF. d4T is inexpensive and widely available but causes mitochondrial toxicity that can lead to sometimes permanent peripheral neuropathy and lipodystrophy. Its phase-out will be difficult given that a large proportion of patients on ART in developing countries are reliant on d4T-containing first line regimens, but the new WHO recommendation may prove to be more cost-effective in the long run [21].

### New Drugs

In addition to updated guidelines, several new antiretroviral (ARV) drugs were approved by the US FDA this past year for treatment-naive HIV patients. Raltegravir, as previously mentioned, was approved based on results from the STARTMRK trial, a double blind controlled study comparing raltegravir to EFV in combination with TDF/FTC [13,22]. Viral suppression at 48 weeks and rate of resistance mutation were comparable, and raltegravir was better tolerated with fewer central nervous system side effects [23].

The use of the CCR5 antagonist maraviroc (Selzentry) was also expanded by the FDA to include ART-naïve patients with CCR5-tropic HIV-1 virus [24]. Maraviroc prevents HIV entry by blocking CCR5 coreceptors. It works well in treatment-naïve patients, most of whom carry CCR5-tropic viruses only. Highly sensitive tropism testing is necessary prior to use since subjects with mixed or CXCR4-tropic HIV-1 infection did not respond well to maraviroc in phase-2 study. The Maraviroc versus Efavirenz Regimens as Initial Therapy (MERIT) trial [25] showed that compared to EFV, maraviroc was slightly less effective at achieving viral suppression below 50 copies/ml in patients with higher baseline VL but was more effective at increasing CD4 counts in ART-naïve patients. Those on maraviroc also reported better lipid profiles. Discontinuation rates were similar among the two groups, though more patients on maraviroc discontinued due to treatment failure, while more patients on EFV discontinued for adverse events. A post hoc analysis of patients screened by a tropism assay with enhanced sensitivity yielded similar results except for fewer discontinuations due to lack of efficacy and a better overall response rate in the maraviroc group, particularly for patients with high baseline VL [26]. Of note, although a previous CCR5-antagonist was suspected of increasing cancer risk in early studies, fewer cases of malignancies were observed in the maraviroc group, but whether or not this difference was statistically significant was not clear.

The development of 2 new pharmaco-enhancing agents, GS9350 [27] by Gilead and SPI-452 [28] by Sequoia pharmaceuticals, represents major steps toward identifying alternatives to ritonavir. Ritonavir is a PI that does not affect HIV VL at the booster dose (100 or 200 mg per day) but alters the metabolism of other PIs through inhibition of the cytochrome P450 3A (CYP3A) enzyme [29]. Ritonavir boosting extends the half-life and increases the maximal concentration achieved by other PIs, allowing more convenient dosing and higher barrier to resistance. However, ritonavir is associated with side effects including dyslipidemia, diabetes, and GI dysfunction [30]. As it is the only validated booster drug used with PIs, Abbott which owns the patent currently has a monopoly in the market. A heat stable formulation is currently accessible in the US and may become more widely available later in 2010, but in many places ritonavir still requires cold-chain storage. Both of the new boosting agents successfully completed phase-2 trials. SPI-452 and GS9350 were both shown to enhance safely and effectively the level of PIs [28,31,32]. In addition, when a fixed dose quad pill containing GS9350, elvitegravir (integrase inhibitor), TDF, and FTC was compared with fixed dose EFV, FTC, and TDF (Atripla), the quad pill had a lower rate of adverse events [32].

## HIV Prevention

### The Hope for an HIV Vaccine

Results from the phase-3 Thai vaccine trial using a combination of ALVAC, a recombinant canarypox vector vaccine, and AIDSVAX, a recombinant glycoprotein-120 subunit vaccine, were released late 2009. Although the two protocol specified analyses (intention-to-treat and per-protocol) only showed a trend toward significance, the modified intention-to-treat analysis excluding 7 subjects who were determined to be HIV-infected at study entry showed a 31% (95% confidence interval 1.1-51.2) reduction in the risk of HIV infection [33-35], making this the first HIV vaccine to have a statistically significant effect. The vaccine did not affect VL or CD4 counts in participants who became infected, and no serious safety concerns were identified. The greatest protective effect was during the first year and in heterosexual participants at low or medium risk. Although the mechanisms by which protection was provided are unknown, the authors concluded that this vaccine may be valuable in a community setting with largely heterosexual risk.

### Prevention with Microbicide Gel

Disappointing news came from the vaginal microbicide gel PRO 2000 trial [36,37] conducted by the Microbicides Development Programme (MDP) from 2005 to 2009 in 9385 women in 4 African countries with high HIV prevalence rates. Women were randomly assigned to receive the PRO 2000 gel (0.5% dose) or placebo, along with free condoms. Despite good adherence and tolerability, no significant difference in infection rates was observed (4.5/100 person-year with the PRO 2000 gel versus 4.3/100 person-year with placebo). A similar but smaller study of 3099 women from 6 sites in Africa and 1 in the US (HPTN035) sponsored by the US National Institutes of Health (NIH) earlier in 2009 was more promising, showing a 30% reduction in HIV infections [38]. This result, however, was just short of the pre-defined criterion for statistical significance. To date, no microbicide has been proven effective in a clinical trial. A TDF-containing microbicide gel is currently being tested as pre-exposure prophylaxis [39]. The CAPRISA-004, a phase-2b study conducted in 980 sexually active women in South Africa compares the efficacy of 1% TDF gel to placebo in preventing HIV infection when used 12 hours before and after intercourse [40,41], and results will be available July 2010.

### Oral ART as Pre-exposure Prophylaxis

As newer drugs with less toxicity have made earlier ART administration feasible, they are also being considered for pre-exposure prophylaxis (PrEP) to prevent HIV infection. TDF was effective in preventing Simian Immunodeficiency Virus (SIV) infection in the macaque model [42], and to date use as PrEP in human trials has revealed no serious safety concerns [43]. Complex mathematical models have shown the potential for oral PrEP to reduce significantly HIV transmission, although its utility and effectiveness have not yet been proven by randomized trials and may be undermined by cost [44], behavioral disinhibition [45], and more frequent transmitted drug resistance [46]. A PrEP trial with TDF/FTC is expected to be completed in late 2010. The iPrEX study, a phase-3 randomized controlled trial, evaluates the efficacy and safety of TDF/FTC (Truvada) among MSM at risk for HIV infection at 11 sites in 5 countries [40].

The Vaginal and Oral Interventions to Control the Epidemic (VOICE) study, a large double blind placebo controlled trial supported by the NIH [39], is underway to test a daily regimen of TDF gel, TDF tablets, or Truvada tablets in up to 5,000 women at risk for HIV infection in four African countries. This innovative study directly compares a microbicide gel with oral tablets and is the first to test a gel that will be applied once daily rather than shortly before sexual intercourse. The safety, efficacy, and acceptability of these approaches will be evaluated. Women comprise of more than half of all people living with HIV, and the majority are infected through heterosexual transmission [47]. If proven effective, this form of PrEP would be vital in circumstances where it is difficult for women to refuse sex or negotiate condom use.

### Prevention with Early ART

HIV transmission is strongly correlated to the concentration of virus in blood (VL), and this is usually reflected in genital secretions [48]. ART significantly reduces HIV transmissibility by reducing VL, which is the basis for treating HIV-infected pregnant women [49], infected partners of sero-discordant heterosexual couples and acutely infected patients to prevent secondary transmissions [50,51].

Universal HIV testing and treatment has also been proposed as a method to control the spread of HIV [52]. Using data from South Africa in a mathematical model, Granich et al predicted that yearly universal voluntary HIV testing with ART provided to all persons testing positive will reduce annual global HIV incidence by 95% to below one per 1000 population per year within 10 years. Within 5 years, the present endemic phase, where most adults living with HIV are not on ART, will transition into an elimination phase, in which most are on ART. Some but not all studies comparing the cost of higher ART demand to the savings from lower HIV transmission, hospitalization, and improved quality of life suggest that such a strategy can be cost-effective in the long run [53-56].

Current evidence is insufficient to support a radical policy change [57], as different models have shown varying degrees of public health benefit from ART depending on the assumptions applied [58,59], and empirical data are lacking. Future randomized trials and operational research must address the potential for over-testing, over-treatment, side effects, resistance, and risk behavior changes. Ethical, human rights, political, and community concerns must be weighed before such a policy can be widely recommended. A delicate balance must be sought especially in settings with scarce health care resources and where patients rely on older generation ARV drugs, which make optimal monitoring and treatment much more challenging.

## HIV Outcomes

### Focus on Non-AIDS Related Deaths

With optimal ART, the life expectancy of persons living with HIV in developed countries approaches that of HIV-negative individuals [60]. As mentioned above, the latest international guidelines emphasize the importance of early therapy. It has also become clear that despite good immune function, ongoing viral replication is injurious and perhaps even accelerates the aging process, by causing persistent immune activation and inflammation [61]. This hypothesis is supported by studies showing that levels of biomarkers for immune activation and inflammation, including C-reactive protein, interleukin-6, and D-dimer, correlate with the use of ART and duration of HIV infection [62,63]. Conditions traditionally considered non-AIDS related, such as cardiovascular, renal, hepatic, and neurologic diseases, as well as certain types of cancers, are becoming the dominant comorbidities in patients on long term ART with CD4 >200cells/mm^3 ^[64]. The cause of non-AIDS related complications is complex and is in part due to adverse effects of long-term ARV use [65]. Cumulative exposure to certain PIs such as lopinavir, indinavir, amprenavir, and fosamprenavir has been associated with increased cardiovascular risk. Some [16,18,65-69] but not all [70-73] observational studies show that risk of myocardial infarction may be raised in patients with current or recent exposure to ABC or didanosine (ddI), although this has not been confirmed by randomized trials [74]. Equally important, chronic HIV replication may mediate changes in the endothelium and clotting and inflammation pathways, causing end organ damage [66,75-77]. As AIDS-related causes of deaths are better controlled with improved ART in developing countries, non-AIDS related comorbidities will become more notable. In the future, adjunct therapies to regulate blood sugar, cholesterol, and other metabolic disturbances will likely play a larger role in managing non-AIDS related comorbidities in resource-limited settings.

### Social Change and Policy

Scientific advances sometimes cannot move society forward without corresponding changes in policy. Worldwide, injection drug use is a major route of transmission for both HIV and viral hepatitis due to needle sharing. Needle exchange programs that provide clean needles to injection drug users (IDUs) are controversial because they are perceived as condoning the illegal and harmful behavior. However, studies have shown that needle exchange decreases transmission of blood borne pathogens and does not increase drug abuse [78]. The US recently lifted a ban on the use of federal funds for needle exchange in recognition of the merit of such programs.

HIV-positive individuals are a vulnerable population not only concerning their health status but also because of social discrimination and stigma. Currently, more than 60 countries have laws that restrict the entry, stay or residence of people living with HIV. In the early 1990's, at the height of the epidemic when effective treatment was still lacking, an entry and immigration policy was passed in the US prohibiting HIV-positive individuals from entering the country. That ban was lifted effective January 2010, sending a clear message combating fear, stigma, and discrimination against people living with HIV and AIDS. As a result, the XIX International AIDS conference will be held in Washington, DC in July 2012.

### Implications for China

Since the scale-up of China's National Free Antiretroviral Treatment Program (NFATP) in 2003, over 80,000 patients have been treated. Nine ARV drugs are currently available, including 6 NRTIs (ABC, ddI, d4T, 3TC, TDF, and AZT), 2 NNRTIs (EFV and NVP), and one boosted PI (LVP/r). In addition, three drugs - raltegravir (integrase inhibitor), darunavir (PI), and etravirine (NNRTI) - are in the process of entering the Chinese market. China's current national guidelines updated in 2008 recommend ART initiation in all patients with CD4 count under 350 cells/mm^3^. The rapid scale-up of HIV treatment and care has led to a dramatic decrease in HIV mortality from roughly 27-30 deaths per 100 person-years before ART to about 4-5 deaths per 100 person-years after ART [79,80].

However, major challenges remain in diversifying and optimizing treatment options provided through the free ART program. d4T is currently used as part of the first-line regimen in close to half of all patients on ART in China [79]. Its total replacement by AZT or TDF as recommended by the WHO will be challenging financially and administratively. However, the long-term cost-effectiveness of the new recommendations should be considered. Bender et al [21] showed that TDF- compared to d4T-based first-line ART can be more durable due to better efficacy and toxicity profiles. Additionally, fixed dose combinations will be essential in reducing pill burden and improving medication adherence [81,82], and newer agents will be needed in special cases such as patients with drug-resistance and IDUs on methadone treatment. Cost is the major barrier to the introduction of new drugs in China, but the NFATP is certainly moving toward the expansion of ARV regimens.

As lower baseline CD4 count correlates to worse prognosis, decreasing the treatment threshold and expanding treatment coverage may be an urgent next step in China to control both AIDS and non-AIDS related mortality and morbidity. Most patients still present with late disease, and fewer than 1 in 3 HIV-positive individuals are aware of their status [83]. At the end of August 2008, the median baseline CD4 count of those enrolling into the NFATP was only 118 cells/mm^3 ^[79]. Resource limitation is the biggest obstacle in implementing more aggressive screening and treatment guidelines. A rise in the number of patients eligible for ART will require increased ARV supplies, improved laboratory monitoring capabilities, and additional trained HIV care providers. The National Center for AIDS/STD Control and Prevention (NCAIDS) will raise funds to ensure sustainable supplies and continue to strengthen the physician education program initiated in 2002. With the expansion of ART coverage, first line drug resistance and second line treatment availability will become bigger concerns. ART resistance testing and surveillance are currently being conducted and will expand to nationwide coverage within the next 5 years. NCAIDS is also considering greater cooperation with nongovernmental organizations (NGOs) as a channel for providing additional adherence counseling. As treated patients live longer, long term ART-related side effects like the above-mentioned mitochondrial toxicities will require improved monitoring and management. Finally, efforts are needed to reduce stigma, which prevents many from receiving HIV testing and treatment. Infection with HIV is often equated with being immoral - it is not uncommon for HIV-positive individuals to be estranged from their family, and the family from the community, all driven by fear and shame. The Chinese government is already taking steps to promote social tolerance by expanding methadone maintenance treatment (MMT) and needle and syringe programs (NSPs), removing the requirement for viral hepatitis testing before school and job entry, and lifting the entry ban on HIV-positive foreigner [84].

Substantial progress has been made in the area of harm reduction, especially among IDUs. In 2006, the Ministry of Health, Ministry of Public Security and the State Food and Drug Administration (SFDA) issued the revised "Opium Abusers Community-Based Drug Maintenance Treatment Protocol," which expanded the MMT program from pilot phase to general application. By the end of 2008, 558 MMT clinics in 23 provinces have served more than 170,000 clients [85]. NSPs have expanded to a total of 790 centers, about half of which were funded by the government as of 2006 [86]. The US NIH also supports an HIV prevention trial among HIV-negative IDUs in the provinces of Guangxi and Xinjiang.

However, major hurdles remain among other risk groups. Sexual transmission has become the fastest growing means of HIV transmission in China due to changing sexual behavior and attitude since China opened up to the outside world in the late 1970s [87]. Among new HIV cases in China in 2007, close to 45% are infected through heterosexual transmission, with the majority being transmission between non-regular partners [88], including female sex workers (FSW) and their clients. In one study, 60% of FSWs in China did not use condoms consistently with their clients [88]. Considerable crossover exists between risk groups, especially among commercial sex workers and IDUs, creating a bridging effect of spreading HIV from IDUs to the clients and regular sex partners of FSWs [89]. While accounting for only about 2-5% of all adult males [90], men who have sex with men (MSM) represent an estimated 12% and rapidly growing proportion of all HIV-positive persons in China [88,91]. The Chinese MSM population is another important bridge for the spread of HIV, as one-half report having sex with women, and one-third being married. Research and intervention among MSM have received increased funding and attention from both the Chinese government and international organizations [92,93]. Since most available studies were conducted in large or medium-sized cities among the well educated, additional research is needed especially in rural areas and among military personnel, prisoners, college students, and migrant workers [94]. For both the FSW and MSM populations, more effective prevention programs should be anchored in empirical evidence-based research. Systematic surveillance and grass root networks must also be strengthened. If proven effective, oral or vaginal forms of PrEP would be useful for sexual partners and uninfected members of these high risk groups. Until then, continual efforts are needed in sexual education, behavior modification, and improved access to HIV testing and counselling.

## Conclusion

We have summarized several recent major themes in HIV/AIDS. We share in the disappointment from setbacks, excitement of new successes, and hope in coming advancements. Future directions in China as well as the rest of the world will continue to focus on methods of HIV prevention such as vaccine and microbicide development, expanding HIV testing and treatment, discovering and developing new ARV drugs, optimizing the ART delivery system, and improving therapy for non-AIDS related conditions and coinfections.

## Competing interests

The authors declare that they have no competing interests.

## Authors' contributions

All authors fulfill the criteria of authorship. KS performed the literature search and was the lead author for the paper. SZ contributed to reviewing the literature as well as planning and drafting the manuscript. RYC, MSC, and FZ carried out critical revision of the manuscript for important intellectual content. All authors read and approved the final manuscript.
